# Near-complete genome sequence of a reassortant fish nervous necrosis virus isolated from a European sea bass (*Dicentrarchus labrax*) in Tunisia

**DOI:** 10.1128/mra.00058-25

**Published:** 2025-09-12

**Authors:** Houda Agrebi, Miriam Abbadi, Alessandra Buratin, Lorena Biasini, Haitham Sghaier, Balkiss Bouhaouala-Zahar, Anna Toffan, Nadia Cherif

**Affiliations:** 1 Disease of Aquatic organism service, Aquaculture Laboratory (LR16INSTM03), National Institute of Sea Sciences and Technologies, Salammbô, Tunisia; 2 Istituto Zooprofilattico Sperimentale delle Venezie, National Reference Centre for Fish, Mollusk and Crustacean Diseases, Legnaro, Italy; 3 Laboratory ″Energy and Matter for Development of Nuclear Sciences″ (LR16CNSTN02), National Center for Nuclear Sciences and Technology (CNSTN) 201296 , Sidi Thabet, Tunisia; 4 Laboratory of Venoms and Therapeutic Molecules (LR16IPT08), Institut Pasteur Tunis, University Tunis El Manar 37964, Tunis, Tunisia; 5 Medical School of Tunis, University Tunis El Manar 37964, Tunis, Tunisia; Katholieke Universiteit Leuven, Leuven, Belgium

**Keywords:** VNN, reassortant, sequencing, sea bass

## Abstract

European seabass (*Dicentrarchus labrax*) from a Tunisian farm showed signs of viral nervous necrosis. Sequencing revealed a reassortant *Betanodavirus* combining RNA1 from *Betanodavirus epinepheli* and RNA2 from *Betanodavirus pseudocarangis*.

## ANNOUNCEMENT

**Fig 1:**
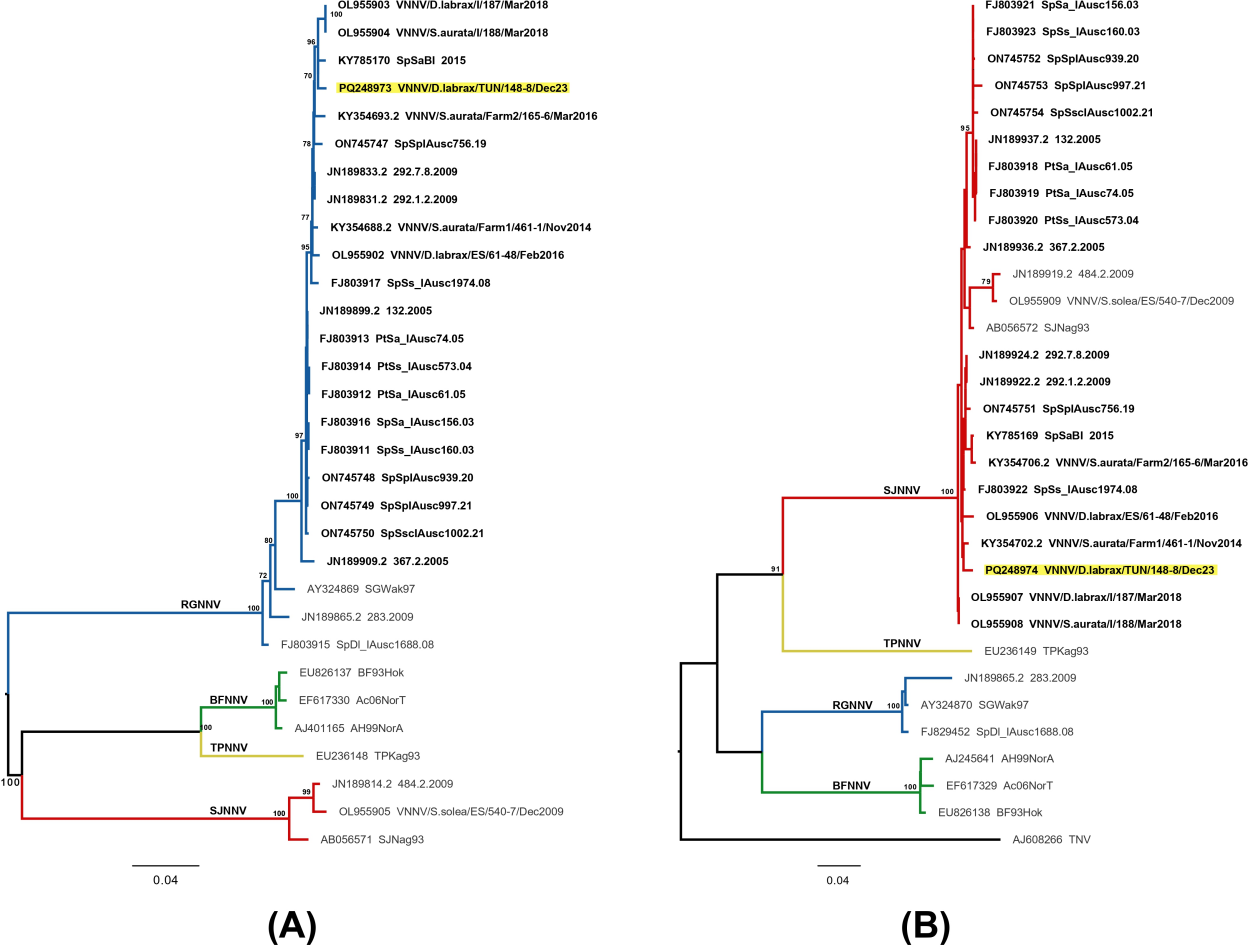
Maximum Likelihood phylogenetic trees generated using the IQ-Tree software v1.6.9 (16), based on RNA1 (**A**) and RNA2 (**B**) nucleotide complete sequences. *Betanodavirus* genotype subdivisions are represented by color-coded branches ● RGNNV- ● BFNNV- ● TPNNV- ● SJNNV. Representative sequences for each genotype are included. The 20 already published sequences from Mediterranean reassortant betanodaviruses are reported in bold. The Tunisian reassortant (RGNNV/SJNNV) strain is highlighted in yellow. The numbers at the nodes represent bootstrap values (only values ≥70% are reported), and branch lengths are scaled according to the number of nucleotide substitutions per site. The scale bar is reported. Phylogenetic trees are visualized with FigTree v1.4 software (http://tree.bio.ed.ac.uk/software/figtree/, accessed on 30 August 2024.

*Betanodavirus,* also known as nervous necrosis virus (NNV), causing the viral encephalopathy and retinopathy disease (VER) in finfish, belongs to the *Nodaviridae* family, genus *Betanodavirus* (1). It is considered a major problem for European seabass (*Dicentrarchus labrax*) farming in the Mediterranean (2). Its linear genome is composed of two positive-sense single-stranded RNA molecules, RNA1 (3.1 Kb) and RNA2 (1.4 Kb), and a third RNA1 subgenomic transcript (RNA3) (0.4 kb) (3–5). Based on the small variable region of RNA2, the following four species have been identified: *Betanodavirus pseudocarangis* (SJNNV), *Betanodavirus takifugui* (TPNNV), *Betanodavirus verasperi* (BFNNV), and *Betanodavirus epinepheli* (RGNNV) (2, 6). Natural reassortment events can occur, and two reassortants (RGNNV/SJNNV and SJNNV/RGNNV) have been described, representing a new challenge for Mediterranean aquaculture (7–11). In the present study, following a mortality event of European seabass in offshore cages located along the Tunisian Sahel coastline in August 2023, NNV was isolated from brain samples using the SSN-1 cell line incubated at 25°C (12). Qualitative real-time reverse transcription polymerase chain reaction (rRT-PCR) targeting RNA1 was performed on the cell supernatant to confirm the viral identity (13). To obtain the complete sequence, the complementary DNA (cDNA) was used as a template in nine different PCRs, five targeting the RNA1 and four targeting the RNA2. The primer sets used for the amplification of the complete genome are detailed in Panzarin et al. (14). cDNA synthesis was carried out using the SuperScriptTM III Reverse Transcriptase Kit, followed by the 9 PCR reactions performed using the Platinum Taq DNA Polymerase kit. Each 25 µL reaction included 5 µL cDNA, 10× buffer, 0.2 mM dNTPs, 1.5 mM MgCl₂, 10 U Taq polymerase, and 0.5 µM of each primer. Expected amplicon sizes were 874, 684, 849, 758, and 903 bp for RNA1, and 593, 605, 424, and 399 bp for RNA2. Amplicons were purified with ExoSAP-IT Express and sequenced bidirectionally using the BrilliantDye Terminator (v3.1) Cycle Sequencing Kit on a 24-capillary ABI PRISM 3500xl Dx Genetic Analyzer. Sequencing data were assembled and edited with SeqScape v3.0. Sequences were aligned and compared to representative ones for each viral species and to all available complete genomes from RGNNV/SJNNV Mediterranean strains using MEGA 7.0 (Table 1) (15). Phylogenetic trees are shown in Fig. 1. The genome obtained was composed of an RNA1 ORF, 2,949 bases long (GC content 52.2%), and an RNA2, measuring 1,023 bases (GC content 55.3%). The RNA1 ORF (982 amino acids) showed high identity (98.9%–99.8%) with the other Mediterranean reassortant strains (nucleic identity: 98.3%–99.1%). The RNA2 ORF (340 amino acids) showed an identity of 98.2%–99.7% (nucleic identity: 97.0%–99.0%).

**TABLE 1:** List of reassortant *Betanodavirus* (RGNNV/SJNNV) strains from the Mediterranean basin used for the phylogenetic analysis^*a*^^,^^*b*^

				GenBank accession no.		
Strain	Host	Country	Year	RNA1	RNA2	Reference
**VNNV/D.labrax/TUN/148-8/Dec23**	***D.labrax***	**Tunisia**	**2023**	PQ248973	PQ248974	**This study**
SpSpIAusc756.19	*S.pilchardus*	Spain	2019	ON745747	ON745751	(17)
SpSpIAusc939.20	*S.pilchardus*	Spain	2020	ON745748	ON745752	(17)
SpSpIAusc997.21	*S.pilchardus*	Spain	2021	ON745749	ON745753	(17)
SpSscIAusc1002.21	*S.scombrus*	Spain	2021	ON745750	ON745754	(17)
VNNV/D.labrax/I/187/Mar2018	*D. labrax*	Italy	2018	OL955903	OL955907	(18)
VNNV/D. labrax/ES/61-48/Feb2016	*D. labrax*	Spain	2016	OL955902	OL955906	(18)
VNNV/S. aurata/I/188/Mar2018	*S. aurata*	Italy	2018	OL955904	OL955908	(18)
SpSaBI 2015	*S. aurata*	Spain	2015	KY785170	MN914162	(19)
VNNV/S. aurata/Farm1/461-1/Nov2014	*S. aurata*	n.d.	2014	KY354688.2	KY354702.2	(10)
VNNV/S. aurata/Farm2/165-6/Mar2016	*S. aurata*	n.d.	2016	KY354693.2	KY354706.2	(10)
132.2005	*D.labrax*	Italy	2005	JN189899.2	JN189937.2	(9)
292.7.8.2009	*D.labrax*	Greece	2009	JN189833.2	JN189924.2	(9)
292.1.2.2009	*S.aurata*	Greece	2009	JN189831.2	JN189922.2	(9)
367.2.2005	*D.labrax*	Italy	2005	JN189909.2	JN189936.2	(9)
SpSa_IAusc156.03	*S.aurata*	Spain	2003	FJ803916	FJ803921	(8)
SpSsIAusc160.03	*S.senegalensis*	Spain	2003	FJ803911	FJ803923	(8)
PtSs_IAusc573.04	*S. senegalensis*	Portugal	2004	FJ803914	FJ803920	(8)
PtSa_IAusc61.05	*S. aurata*	Portugal	2005	FJ803912	FJ803918	(8)
PtSa_IAusc74.05	*S. aurata*	Portugal	2005	FJ803913	FJ803919	(8)
SpSs_IAusc1974.08	*S. senegalensis*	Spain	2008	FJ803917	FJ803922	(8)

^*a*^ The following information is reported: strain identification code, host, country, year of identification, GenBank accession numbers for RNA1 and RNA2 complete genomes, and references. n.d. = not disclosed.

^*b*^ Bold values indicates the newly Tunisian sequenced RNA 1 and RNA2 in this study.

Phylogenetic analysis demonstrated that RNA1 clustered with RGNNV (Fig. 1A) and RNA2 with SJNNV genotype (Fig. 1B). To date, only 20 complete genome sequences from RGNNV/SJNNV viruses are available, and only five are from *D. labrax* and all come from European countries. In 2019, an RGNNV/SJNNV was detected for the first time in seabream larvae in Tunisia (20), and herein, we report the reassortant RGNNV/SJNNV full genome obtained from European seabass in Tunisia.

## Data Availability

Generated sequences are available in GenBank under the accession numbers PQ248973 (RNA1) and PQ248974 (RNA2).
